# Supplementary material for: MetaRibo-Seq measures translation in microbiomes
Source: Nat Commun. 2020 Jun 29;11:3268. doi: 10.1038/s41467-020-17081-z (PMC7324362; doi:10.1038/s41467-020-17081-z)
Supplement: Supplementary file 10 — Supplementary Data 7 [file 41467_2020_17081_MOESM10_ESM.zip › File2/Confidence_VeryHigh_Taxonomy/226628_out.krona.html]

Javascript must be enabled to view this page.

members
magnitude
magnitudeUnassigned
count
unassigned
taxon
rank

226628\_out

14

14
2
superkingdom

1239
phylum
14

class
186801
14

14
order
186802

13
186803
family

1
572511
genus

species
40520

SRS018623\_contig\_number\_contig-100\_2260.49783
1

33042
genus
12

SRS021484\_contig\_number\_contig-100\_836.836SRS064276\_contig\_number\_contig-100\_5286.230008SRS104636\_contig\_number\_contig-100\_24810.116664SRS150029\_contig\_number\_contig-100\_15843.91151SRS893295\_contig\_number\_contig-100\_34291.34292
5

2049024
species
1

SRS1041145\_contig\_number\_23895


SRS049446\_contig\_number\_11915SRS098073\_contig\_number\_13045SRS1055022\_contig\_number\_12644SRS147139\_contig\_number\_26333SRS149325\_contig\_number\_16286
5
species
1896997

species
33043

SRS019068\_contig\_number\_48915
1

186806
family
1

genus
1730
1

1

SRS019068\_contig\_number\_33099
1262889
species
